# Natural Killer Cell Infiltration in Prostate Cancers Predict Improved Patient Outcomes

**DOI:** 10.1038/s41391-024-00797-0

**Published:** 2024-02-28

**Authors:** Nicholas A. Zorko, Allison Makovec, Andrew Elliott, Samuel Kellen, John R. Lozada, Ali T. Arafa, Martin Felices, Madison Shackelford, Pedro Barata, Yousef Zakharia, Vivek Narayan, Mark N. Stein, Kevin K. Zarrabi, Akash Patnaik, Mehmet A. Bilen, Milan Radovich, George Sledge, Wafik S. El-Deiry, Elisabeth I. Heath, Dave S. B. Hoon, Chadi Nabhan, Jeffrey S. Miller, Justin H. Hwang, Emmanuel S. Antonarakis

**Affiliations:** 1 Masonic Cancer Center, University of Minnesota-Twin Cities, Minneapolis, MN USA; 2 Caris Life Sciences, Phoenix, AZ USA; 3 University Hospital Seidman Cancer Center, Cleveland, OH USA; 4 Holden Comprehensive Cancer Center, Iowa City, IA USA; 5 Abramson Cancer Center, University of Pennsylvania, Philadelphia, PA USA; 6 Herbert Irving Comprehensive Cancer Center, Columbia University New York, New York, NY USA; 7 Sidney Kimmel Cancer Center, Jefferson Medical College, Philadelphia, PA USA; 8 University of Chicago Medicine Comprehensive Cancer Center, Chicago, IL USA; 9 Winship Cancer Institute of Emory University, Atlanta, GA USA; 10 Legorreta Cancer Center, Brown University, Providence, RI USA; 11 Karmanos Cancer Institute, Wayne State University, Detroit, MI USA; 12 Saint John’s Cancer Institute, Saint John’s Health Center PHS, Santa Monica, CA USA

**Keywords:** Cancer therapy, Cancer genetics, Prognostic markers

## Abstract

**Background:**

Natural killer (NK) cells are non-antigen specific innate immune cells that can be redirected to targets of interest using multiple strategies, although none are currently FDA-approved. We sought to evaluate NK cell infiltration into tumors to develop an improved understanding of which histologies may be most amenable to NK cell-based therapies currently in the developmental pipeline.

**Methods:**

DNA (targeted/whole-exome) and RNA (whole-transcriptome) sequencing was performed from tumors from 45 cancer types (*N* = 90,916 for all cancers and *N* = 3365 for prostate cancer) submitted to Caris Life Sciences. NK cell fractions and immune deconvolution were inferred from RNA-seq data using quanTIseq. Real-world overall survival (OS) and treatment status was determined and Kaplan–Meier estimates were calculated. Statistical significance was determined using X^2^ and Mann–Whitney *U* tests, with corrections for multiple comparisons where appropriate.

**Results:**

In both a pan-tumor and prostate cancer (PCa) -specific setting, we demonstrated that NK cells represent a substantial proportion of the total cellular infiltrate (median range 2–9% for all tumors). Higher NK cell infiltration was associated with improved OS in 28 of 45 cancer types, including (PCa). NK cell infiltration was negatively correlated with common driver mutations and androgen receptor variants (*AR-V7*) in primary prostate biopsies, while positively correlated with negative immune regulators. Higher levels of NK cell infiltration were associated with patterns consistent with a compensatory anti-inflammatory response.

**Conclusions:**

Using the largest available dataset to date, we demonstrated that NK cells infiltrate a broad range of tumors, including both primary and metastatic PCa. NK cell infiltration is associated with improved PCa patient outcomes. This study demonstrates that NK cells are capable of trafficking to both primary and metastatic PCa and are a viable option for immunotherapy approaches moving forward. Future development of strategies to enhance tumor-infiltrating NK cell-mediated cytolytic activity and activation while limiting inhibitory pathways will be key.

## Introduction

Metastatic castration-resistant prostate cancer (mCRPC) remains a significant burden and a leading cause of death for men in the United States and throughout the world. While there has been significant interest in harnessing the immune system to treat mCRPC [1–3], only a small proportion of patients derive a benefit. Currently approved immunotherapies include Sipuleucel-T [4] and pembrolizumab, which is approved for only a narrow subset of patients with tumor mutation burden high features or microsatellite instability (~3–5% of mCRPC patients) [5, 6]. Attempts using other immune checkpoint inhibitors in isolation and in combination have had mixed results in small subsets of patients and remains under exploration [7–11]. There remains an urgent need to develop immune and cellular-based therapies that are both efficacious and applicable to a broader population. Numerous T cell products have been either developed or are under development for mCRPC, including chimeric antigen receptor (CAR) T cells and T cell engagers as reviewed by Zarrabi et al. [12]. Similar products are under development utilizing natural killer (NK) or NK-T cells as the effectors [13–15] as previously reviewed by our group [16].

Natural killer cells are cytotoxic lymphocytes which are part of the innate immune system and are capable of destroying malignant and virally-infected cells without the need for priming as is required by T cells [17]. Circulating NK cells comprise between 5 and 20% of lymphocytes and the vast majority are phenotypically-defined by the presence of the surface marker CD56 and absence of CD3 (CD56+/CD3−) with variable CD16 expression depending on developmental stage. Notably, there are subsets of CD56 negative NK cells [18], which are missed by conventional analysis for this marker and has led to the proposal of alternative markers such as NKp46 and NKp30 [19]. NK cells are not antigen-specific [20]; however, NK cells can be redirected with CARs [21, 22] or via immune engagers/monoclonal antibodies to induce antibody-dependent cellular cytotoxicity (ADCC) [23]. In contrast to T cell-derived immune therapies, NK cell approaches allow for use of allogeneic (off-the-shelf) products, do not cause graft-versus-host disease, and have considerably lower risk for cytokine release syndrome (CRS) and immune effector cell-associated neurotoxicity syndrome (ICANS) [24]. Due to these key characteristics, development of NK cell products and strategies to overcome dysfunction are of increasing interest [25].

No prior studies have delved into the influence of tumor-intrinsic mutational and gene expression profiles [26] associated with NK cell infiltration and the association with outcomes with standard-of-care (SOC) therapies used for treatment of PCa. Using the largest collection of PCa samples with comprehensive molecular profiling of DNA and RNA (*n* = 3365) from a database of real-world patient samples, we sought to evaluate whether NK cells infiltrate into PCa, to explore effects of NK cell infiltration on patient outcomes, and to understand tumor features affecting NK cell infiltration.

## Materials and methods

### Specimens

We retrospectively reviewed molecular alterations and related survival outcomes of *N* = 90,916 for all cancers and *N* = 3365 for PCa. Comprehensive molecular profiling, including whole-exome sequencing (WES) and whole transcriptome sequencing (WTS), was performed in a CLIA/CAP/ISO15189 certified clinical laboratory (Caris Life Sciences, Phoenix, AZ, USA) [27]. This study was conducted in accordance with the guidelines of the Declaration of Helsinki, Belmont Report, and U.S. Common Rule. In keeping with 45 CFR 46.101(b)(4), this study was performed by using retrospective and deidentified clinical data. Therefore, this study was considered institutional review board exempt, and no patient consent was necessary.

### Survival analysis

Real-world evidence (RWE) outcomes were assessed from insurance claims data. RWE overall survival (OS) was defined as time of treatment initiation date to either death or last contact in the insurance claims repository. As previously reported, patient death was assumed for any patient without a claim for more than 100 days, which holds true for more than 95% of patients with a recorded death in the NDI (National Death Index). Cox proportional hazard ratios were calculated for each comparison group and significance was determined as *p* values of <0.05 using the log-rank statistic.

### DNA Next-generation sequencing (NGS)

Direct sequence analysis was conducted on genomic DNA isolated from microdissected, formalin-fixed, paraffin-embedded tumor samples using the NextSeq platform (Illumina, Inc., San Diego, CA) for 592 cancer-relevant genes [28] or the Illumina Novaseq 6000 platform for whole exome sequencing (WES). For WES, a hybrid pulldown of baits designed to enrich for 720 clinically relevant genes at high coverage and high read-depth was used, along with another panel designed to enrich for an additional >20k genes at a lower depth and a 500 Mb SNP backbone panel (Agilent Technologies) to help with gene amplification/deletion detection as previously described [27].

### RNA whole transcriptome sequencing (WTS)

Qiagen RNA FFPE tissue extraction kit was used for extraction. RNA quality and quantity were determined using the Agilent TapeStation (Agilent TapeStation Laptop, RRID:SCR_019547). Biotinylated RNA baits were hybridized to the synthesized and purified cDNA targets, and the bait–target complexes were amplified in a post-capture PCR reaction. The Illumina NovaSeq 6500 was used to sequence the whole transcriptome from patients to an average of 60 M reads. Raw data was demultiplexed by the Illumina Dragen BioIT accelerator, trimmed, counted, removed of PCR-duplicates, and aligned to human reference genome hg19 by the STAR aligner. For transcription counting, transcripts per million molecules were generated using the Salmon expression [27].

### Immune cell infiltration

To examine the associations with NK cells in PCa patients, NK cell tumor infiltration was inferred using quanTIseq, a computational method for quantifying immune cell fractions by deconvolution of bulk RNA-seq data [29]. NK cell infiltration was assessed in 90,916 tumor biopsies across 45 distinct tumor types, including 3365 PCa patients.

### Statistical analyses

Statistical significance was determined using X^2^ and Mann–Whitney *U* tests with corrections for multiple comparisons (Benjamini-Hochberg) where appropriate.

## Results

### Characterization of NK cell fractions in PCa

**Fig. 1:**
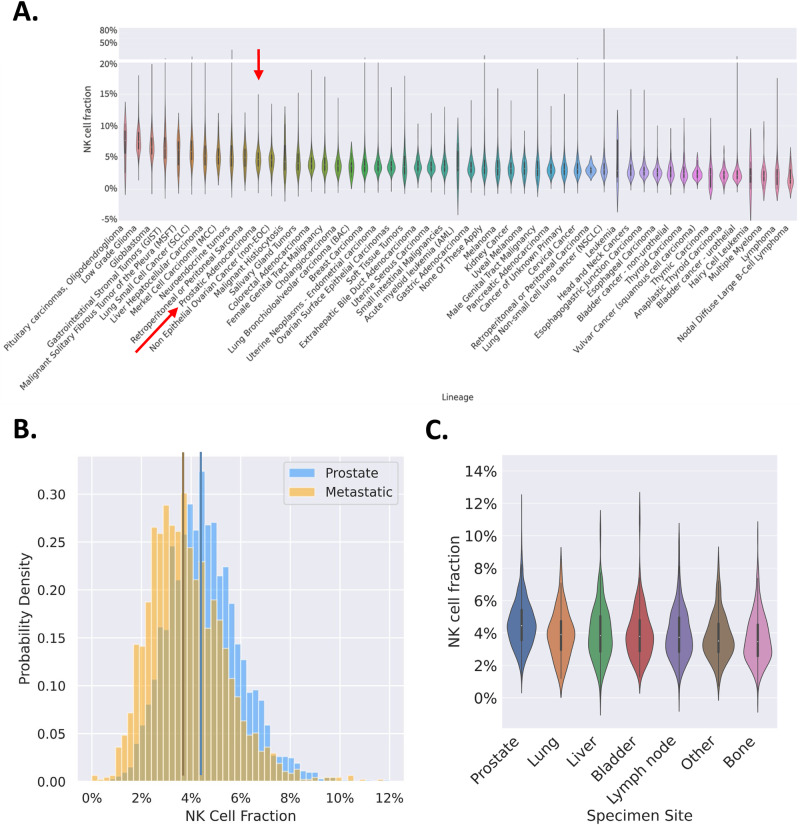
Characterization of NK cells in tumors. **A** NK cell fraction as a percentage of all cells calculated using quanTiseq for immune deconvolution using transcriptomic data. A total of 90,916 samples from 45 distinct tumor types were analyzed from a commercial database of real-world patient samples. Data shown in violin plots in which the white dot represents the median and the black box shows the ends of the first and third quartiles. *p* * < 0.05, ** < 0.01, *** < 0.001, **** < 0.0001. **B** Distribution of quanTIseq NK cell score across PCa tumors from the prostate and metastatic PCa tumors. Vertical dotted lines represent the median NK cell fraction for each subgroup. **C** Metastasis was then divided by anatomical sites, including liver and bone. Cohorts shown are stratified by the median NK cell fraction. Distribution of NK cell scores shown in violin plots in which the boundary of the violin represents the range of all data points.

NK cell infiltration was assessed in 90,916 tumor biopsies across 45 distinct tumor types, including 3,365 PCa patients. Median NK cell fraction by tumor type ranged from a high of 7–9% (medulloblastoma and gliomas) to a low of 2% (thyroid and thymic cancers), with a pan-cancer median of 3.50% (95% CI 3.49–3.51%) (Fig. 1A). Based on these measurements, PCa had the 11th highest median NK cell fraction among the 45 cancer types examined (4.9%, 95% CI 4.8–5.0%). Given that prostate tumors have distinct outcomes and molecular characteristics based on disease site, we also compared the distribution of NK cell fractions between PCa biopsies from prostate, and distant metastatic sites (Fig. 1B). In tumors from metastatic PCa (mPCa) sites, the NK cell fractions were lower (4.1%, 95% CI 4.0–4.2%) compared to prostate biopsy samples. We also observed notable NK cell fractions upon partitioning the samples into common metastatic sites including the liver, bone, lymph node, lung, and bladder (Fig. 1C). Available demographic data for groups are included in Table S1.

### NK cell profiles are associated with overall clinical outcomes and treatment regimen

**Fig. 2:**
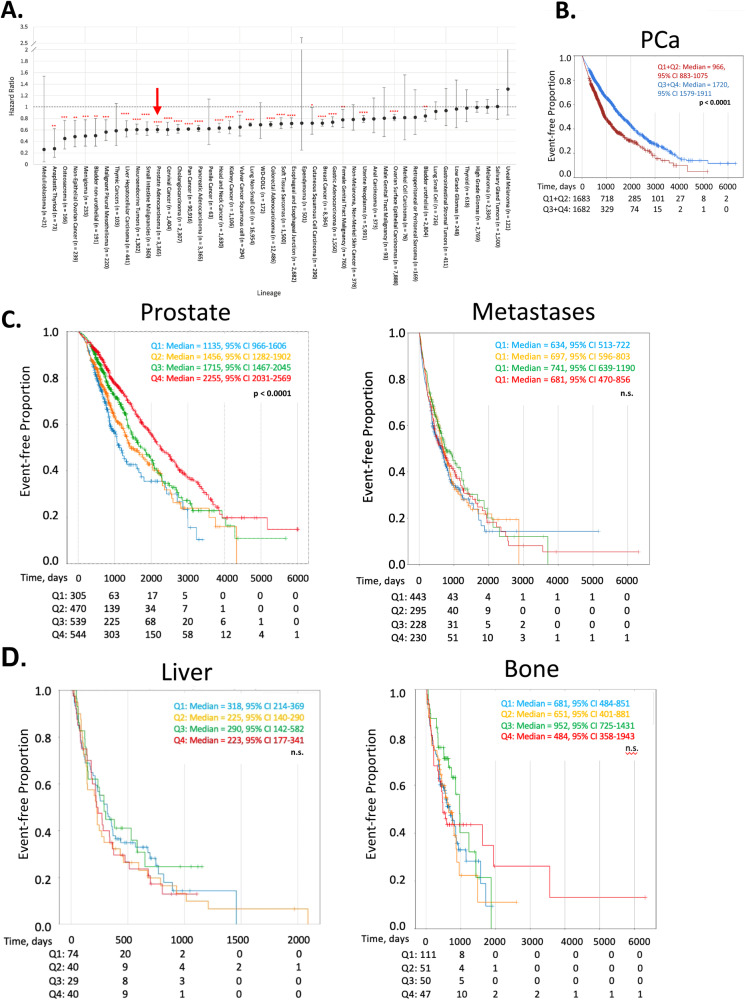
NK cell profiles association with OS. **A** Association of overall survival (OS) in tumors with top 75th percentile NK cell infiltration (relative to all cancers) across 45 different tumor types. Dotted line represents the Hazard Ratio of 1.0. *p* * < 0.05, ** < 0.01, *** < 0.001, **** < 0.0001. **B** Progression-free survival (PFS) of patients with high (>= median) NK infiltration compared to low (<median) NK infiltration in 45 distinct tumor types. **C** OS of patients with tumors from the prostate and metastasis were partitioned into 4 quartiles as defined by the relative NK cell abundance. **D** OS for metastasis was further divided by common anatomical metastatic sites, including liver and bone. Cox proportional hazard ratios were calculated for each comparison group with significance determined as *p* values of <0.05 using log-rank statistics.

Within each cancer type, we stratified samples into NK cell-low and -high subgroups (<50th vs >50th percentile, respectively). For 28 of 45 cancer types that we examined, high levels of NK cell fractions were associated with improved OS (HR for individual tumor types ranging from 0.28–0.84, *p* < 0.05), while 16 of 45 cancer types (including low-grade glioma, GIST, and thyroid cancers) had HR < 1.0 (range from 0.264–0.997) with 95% CI crossing 1.0 (Fig. 2A). Among all PCa patients, NK cell-high tumors were associated with significantly improved OS (Fig. 2B, HR 0.64, 95% CI 0.57–0.71, *p* < 0.00001). We further stratified the PCa patients by NK cell fraction quartiles to assess more granular associations with clinical outcomes. Increased NK cell fractions were associated with improved OS in PCa patients who had undergone primary prostate biopsy (Fig. 2C). However, NK cell infiltrates were not associated with statistically significant differences in outcomes regardless of metastatic biopsy site (Fig. 2D).

Interestingly, biopsies derived from the prostate with high NK fractions exhibited improved outcomes when treated with ADT (*p* < 0.0001), there was no significant difference in OS from the start of treatment among patients with metastatic biopsy samples (Fig. S1A), but no association with docetaxel (Fig. S1B). Similar to outcomes of patients treated with ADT, we observed that high levels of NK cell infiltrates were significantly associated with anti-PD(L)1 response (*p* = 0.04) in samples derived from the prostate (Fig. S1C).

### The interaction of NK cell profiles with driver mutations in PCa

**Fig. 3:**
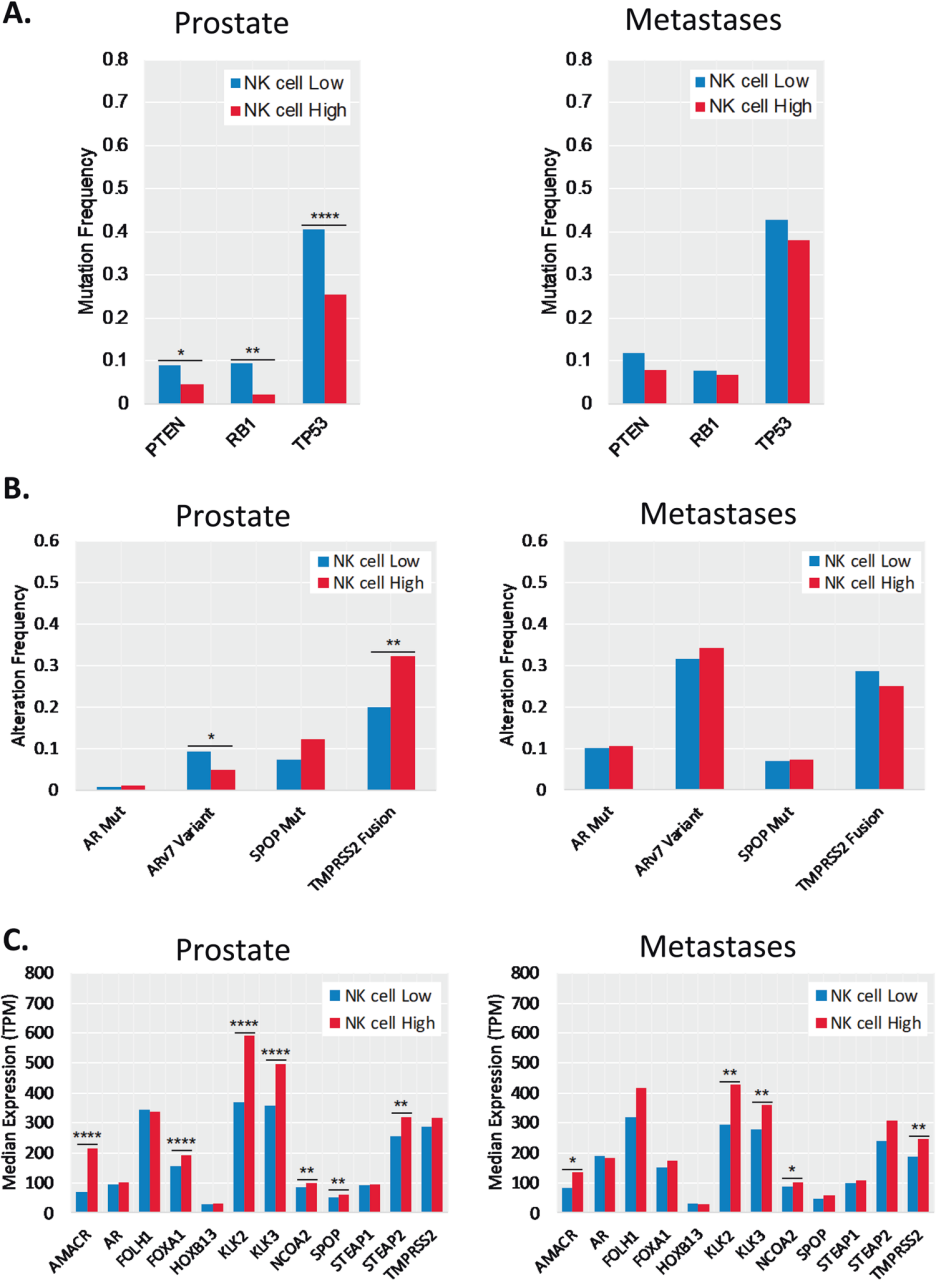
Interaction of NK cell profiles with driver mutations in PCa. Pathogenic mutation frequency of common PCa drivers (**A**), median WTS (TPM) for AR associated genes (**B**), and pathogenic mutation frequency for AR associated genes (**C**) across NK cell high and low prostate and metastatic samples. *p* * < 0.05, ** < 0.01, *** < 0.001, **** < 0.0001. Cox proportional hazard ratios were calculated for each comparison group with significance determined as *p* values of <0.05 using log-rank statistics.

To understand how NK cells may be associated with specific drivers or pathogenic mutations in PCa, we compared the mutation and mRNA expression profiles of NK cell-low (Q1) and -high (Q4) populations. Recurrent genomic alterations in *RB1*, *TP53*, and *PTEN* are associated with advanced metastatic PCa and poor prognosis [30–32]. For both prostate and metastatic biopsies, we observed significantly lower *RB1*, *TP53*, and *PTEN* mutation frequencies in NK cell-high tumors relative to NK-low tumors (Fig. 3A). AR alterations, including genomic alterations, aberrant expression of splice variants (e.g. *AR-V7*), and genes that regulate AR signaling are key drivers of advanced PCa and poor hormonal responsivenness [30–32]. Interestingly, we found that *TMPRSS2-ERG* fusions were significantly more common in NK cell-high tumors from both the prostate, but not metastatic biopsies. *AR-V7* expression was significantly decreased in NK cell-high tumors from prostate biopsies (Fig. 3B). Examining the expression of *AR* and *AR*-related signaling genes, we found that key targets such as *KLK2*, *KLK3*, *STEAP2*, and *FOXA1* demonstrated increased expression in NK-high samples even without increases in *AR* expression (Fig. 3C). Altogether, while *AR* transcriptional targets indicate activated AR signaling in NK cell high tumors, the activity was not consistently associated with higher levels of *AR or AR-V7*.

To analyze for effects in other signaling pathways, we conducted Gene Set Enrichment Anlaysis (GSEA) on the transcription profiles of the tumors either from the prostate, or metastatic sites [33]. Overall, there were no Hallmark pathways enriched in the NK cell high samples, regardless of whether the sample was obtained from the prostate or a metastatic site. However, several pathways were enriched in the NK cell low samples, which was reflected in the negative Normalized Enrichment Scores (NES) (Table S2). We additionally included the differential expression profiles in Table S3. Of specific pathways that have been implicated in immune or tumor cell regulation, there was not enrichment of pathways that reflect cell cycle regulation, apoptosis, senescence, or proliferation. We found that the epithelial to mesenchymal transition (EMT) signature had a negative NES, and thus was enriched in NK low samples.

### NK cell abundance and current immune checkpoint inhibitor (ICI) targets

**Fig. 4:**
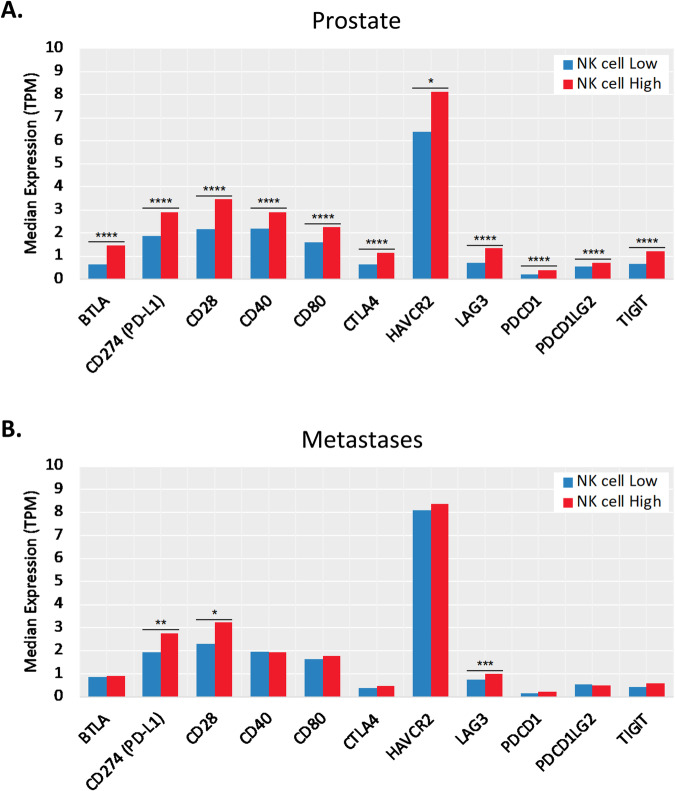
NK cell association with current ICI targets. Median WTS (TPM) for immune regulatory genes, some of which are immunotherapy targets, across NK cell high and low (**A**) prostate and (**B**) metastatic samples. *p* * < 0.05, ** < 0.01, *** < 0.001, **** < 0.0001. Cox proportional hazard ratios were calculated for each comparison group with significance determined as *p* values of <0.05 using log-rank statistics.

To determine whether NK cell infiltrates are associated with these immunomodulatory receptors, we examined the expression levels of pro- and anti-inflammatory immune regulators in NK-high and -low prostate and metastatic PCa tumors (Fig. 4). In biopsies from the prostate, high NK cell infiltration was associated with a significant increase in all analyzed target genes, including T cell-expressed costimulatory marker CD28 and professional antigen presenting cell-expressed second signals CD40 and CD80. There was concurrent and significantly increased expression of the immune checkpoint pathway markers *HAVCR2* (*TIM-3*), *CTLA-4*, *PDCD1 (PD-1), PDCD1LG2 (PD-L1)*, *LAG3*, and *TIGIT*, demonstrating increased immune suppressive signaling from both the tumor and immune cell populations. Metastatic biopsy samples had increased expression of *PD-L1*, *CD28*, and *LAG3* (Fig. 4).

### Interaction with other immune cells and chemokines

**Fig. 5:**
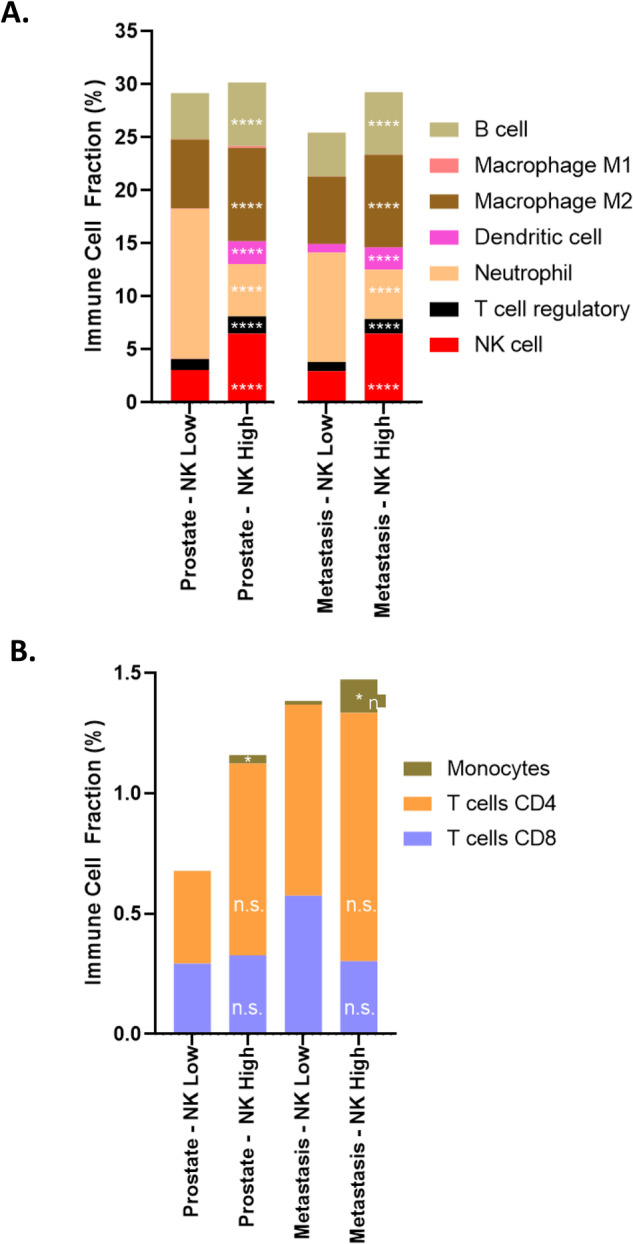
NK interaction with other Immune cells. **A** Immune cell deconvolution using quanTiseq demonstrates differences in cell fraction of B cells, macrophage M1/ M2, dendritic cells, neutrophils, T cell regulatory, and NK cells, as well as (**B**) monocytes, and T cells CD4/ CD8 in prostate and metastatic PCa tumors when comparing Q1 and Q4 NK cell infiltration groups. *p-values (**** < 0.0001).

We next characterized the TME composition of NK cell-high and -low samples. Primary prostate tumors with high NK cells had significantly increased B cell and M2 macrophage infiltration (1.4-fold increase, *p*-value < 0.0001), while neutrophil infiltration was significantly decreased compared to NK cell-low tumors (2.8-fold decrease, *p*-value < 0.0001) (Fig. 5). In PCa tumors from metastatic sites, there were similar increases in B cell and M2 macrophage counts in NK cell-high tumors (1.4-fold increase, *q* < 0.0001 for both) (Fig. 5). Neutrophil infiltration was decreased in metastatic NK cell-high tumors as well, but to a lesser extent than tumors from the prostate (2.2-fold decrease, *q* < 0.0001) (Fig. 5). NK cell-high tumors additionally had significant increases in myeloid dendritic cell infiltration in both primary PCa (10.87-fold increase, *q* < 0.0001) and metastatic biopsy samples (1.38-fold increase, *p*-value < 0.0001) (Fig. 5). Although the effects were greater in the prosatate samples, both NK cell-high PCa tumors from the prostate and metastatic sites exhibited significant association with the expression of a consistent set of chemokines including *CCL19* and CCL6 (Fig. S2). *CX3CL1* and *CXCL3* were increased in primary prostate biopsies only (Fig. S2).

## Discussion

In this study we present the largest compilation to our knowledge of whole exome and whole transcriptome analyses from PCa patients, consisting of 3365 PCa specimens. The prior largest datasets analyzed from The Cancer Genome Atlas (TCGA) contained 497 [34] and 537 [35] PCa samples, respectively. It is important to note that the vast majority of cancer samples within TCGA are pre-treatment primary prostate tumors, while our cohort is a mix of pre- and on-treatment samples obtained from the prostate and prevalent metastatic sites such as the liver and bone [36]. Within PCa itself, we demonstrated robust infiltration of NK cell populations of approximately 5% of the total cell number. We further showed that PCa was among the top tumor types in terms of NK cell infiltration, pointing towards the possibility that NK cell therapies may be more useful than previously thought for this cancer type. Due to differences in output (percent of total cells [35] and cell score [34]), it is difficult to make direct comparisons between our study and those previously published.

When we further stratified OS with the biopsy site, the upper quartile of NK cell infiltration was associated with statistically significant prolongation of survival for prostatic samples. No survival benefit was noted in patients from whom metastasic samples were sequenced. We then sought to evaluate whether the specific treatment regimen utilized had an effect upon overall survival based on quartiles of NK cell infiltration and biopsy site. Survival was improved in patients with upper quartile NK cell infiltration who underwent prostate biopsy and treatment with ADT. Note that we were not able to further stratify based on receipt of novel androgen receptor targeting (ART) versus conventional ADT.

Significantly reduced NK cell infiltration was associated with the presence of known PCa driver mutations *PTEN*, *RB1*, or *TP53* in primary biopsy samples. We then focused on pathognomonic mutations associated with PCa and discovered that *AR-V7* variants were more frequently associated with lower NK cell infiltration, while good-prognosis *TMPRSS2-ERG* fusions [37–40] were associated with increased NK cell infiltration in primary prostate samples. Whether these associations with NK cell infiltration were driven by better prognosis at baseline or if there is a role for ADT in NK cell function and infiltration remain to be determined.

In both primary prostate and metastatic biopsies, markers of immune stimulation/co-stimulation (*CD28*) and exhaustion (*LAG3* and *PD-L1*) were significantly increased in NK cell-high samples. While our evidence thus far points towards improved survival of patients with increased NK cell function, our analysis of these markers demonstrates that the NK cells that are infiltrating into the prostate are likely either exhausted or become quickly exhausted, further limiting their ability to destroy prostate cancer cells that they encounter. Of particular interest is the marked reduction in immunosuppressive neutrophils, M2 macrophages, and regulatory T cells, which is consistent with a compensatory regulatory mechanism counteracting increased inflammation induced by infiltrating NK cells [41–43]. Interestingly, CD4 and CD8 populations were not significantly associated with differences in NK cell infiltration.

Finally, we sought to determine possible differences in chemokines that may be affecting NK cell infiltration into prostate tissue. Across both primary prostate and metastatic biopsies, *CCL19* and *CXCL6* were elevated in NK cell-high samples, while *CCL5*, *CXCL1* and *CX3CL1* were increased only in primary prostate biopsies. CCL19 is primarily a chemoattractant for activated CD56^bright^ NK cells and does not induce cytotoxicity [44]. CCL5 is typically associated with dendritic, T, and resting NK cell chemotaxis while augmenting NK cell cytolytic activity [44]. This is in contrast to resting CD56^dim^ NK cells which typically lack CC family chemokine receptors and would not be affected by CCL5 [44]. CCL5 is a particularly important factor for stimulation of NK cell cytolytic activity and is produced by activated NK cells. CXCL1 is bound by CXCR2 and highly produced by neutrophils, while promoting trafficking of immune suppressive myeloid cells [45]. Our associations strongly support the presence of chemokines that both attract NK cells to the PCa TME and induce NK activation.

There are limitations to our study given the nature of the samples and bulk RNA sequencing methods used. While tissues were microdissected to enrich for tumor tissue, we are limited in describing the immune landscape of these tumor samples due to the use of bulk sequencing rather than single-cell (sc) RNA sequencing, although scRNA datasets remain somewhat limited in the number of samples analyzed [46, 47]. Thus, the immune populations in this study are inferred based on gene expression signatures of the whole microdissected sample rather than from individual cells. Different methods for immune cell deconvolution of RNA-seq data have been utilized, with CIBERSORT [48] being the most common method used in comparable studies and quanTIseq being used in ours [29]. quanTIseq (quantification of the Tumor Immune contexture from human immune RNA-seq data) is a computational method that determines the abolute fractions of immune cells using unique bulk gene expression signatures based on 10 distinct immune cell types [29]. Altought this is used on bulk WTS data, quanTIseq outputs have been validated compared to flow cytometry, immunohistochemistry, and multiple publicly available data sets [29]. Direct comparisons between data produced from these two analytic tools (CIBERSORT versus quanTIseq) are difficult, but Finotello et al. [49] did compare various immune deconvolution strategies to gold-standard FACS and immunohistochemistry. That study demonstrated an overall strong correlation between bulk RNA-seq data analyzed with immune deconvolution strategies and gold-standard analysis with typical r-values of >0.68 for macrophages/monocytes, NK cells and total CD4 + T cells [49].

Due to the nature of the biopsies collected, it is not possible to make inferences regarding associations with localized versus metastatic disease as well or include clinical features such as Gleason score due to the information included with our dataset, limiting further correlative analyses. In particular, prostate biopsies are not necessarily solely from patients with localized disease as the prostate may be biopsied even in metastatic cases; however, we can make inferences regarding metastatic disease alone.

## Conclusions

We present here the largest study analyzing the immune environment and associated molecular features associated with NK cell infiltration in prostate adenocarcinoma, strongly supporting further development of NK cell therapies to treat prostate cancers. Strategies to reduce immune populations that suppress cytotoxic NK cells from infiltrating prostate cancer tissue are needed along with molecular markers or engagers to both promote NK cell infiltration and prevent NK cell exhaustion. These results point to the potential promise of NK cell therapies, including bi- and tri-specific immune engager molecules and CAR-NK, iNK-T therapies for the treatment of PCa.

## Supplementary information

Supplemental Figure 1

Supplemental Figure 2

Supplemental Table 1

Supplemental Table 2

Supplemental Table 3

Supplemental Figure Legends

## Data Availability

The datasets analyzed during the current study are not publicly available but can be made available upon reasonable request. The deidentified sequencing data are owned by Caris Life Sciences, and qualified researchers can apply for access by contacting Caris Life Sciences and signing a data usage agreement.
