# Correction: Natural killer cell infiltration in prostate cancers predict improved patient outcomes

**DOI:** 10.1038/s41391-025-01009-z

**Published:** 2025-08-12

**Authors:** Nicholas A. Zorko, Allison Makovec, Andrew Elliott, Samuel Kellen, John R. Lozada, Ali T. Arafa, Martin Felices, Madison Shackelford, Pedro Barata, Yousef Zakharia, Vivek Narayan, Mark N. Stein, Kevin K. Zarrabi, Akash Patnaik, Mehmet A. Bilen, Milan Radovich, George Sledge, Wafik S. El-Deiry, Elisabeth I. Heath, Dave S. B. Hoon, Chadi Nabhan, Jeffrey S. Miller, Justin H. Hwang, Emmanuel S. Antonarakis

**Affiliations:** 1 Masonic Cancer Center, University of Minnesota-Twin Cities, Minneapolis, MN USA; 2 Caris Life Sciences, Phoenix, AZ USA; 3 University Hospital Seidman Cancer Center, Cleveland, OH USA; 4 Holden Comprehensive Cancer Center, Iowa City, IA USA; 5 Abramson Cancer Center, University of Pennsylvania, Philadelphia, PA USA; 6 Herbert Irving Comprehensive Cancer Center, Columbia University New York, New York, NY USA; 7 Sidney Kimmel Cancer Center, Jefferson Medical College, Philadelphia, PA USA; 8 University of Chicago Medicine Comprehensive Cancer Center, Chicago, IL USA; 9 Winship Cancer Institute of Emory University, Atlanta, GA USA; 10 Legorreta Cancer Center, Brown University, Providence, RI USA; 11 Karmanos Cancer Institute, Wayne State University, Detroit, MI USA; 12 Saint John’s Cancer Institute, Saint John’s Health Center PHS, Santa Monica, CA USA

**Keywords:** Cancer therapy, Cancer genetics, Prognostic markers

Correction to: *Prostate Cancer and Prostatic Diseases* 10.1038/s41391-024-00797-0, published online 28 February 2024

In this article, the author name Akash Patnaik was incorrectly written as Akash Patniak.

The original article has been corrected.

