# Supplementary material for: Natural Killer Cell Infiltration in Prostate Cancers Predict Improved Patient Outcomes
Source: Prostate Cancer Prostatic Dis. 2024 Feb 28;28(1):129–37. doi: 10.1038/s41391-024-00797-0 (PMC11349934; doi:10.1038/s41391-024-00797-0)
Supplement: Supplementary file 1 — Supplemental Figure 1 [file 41391_2024_797_MOESM1_ESM.pdf]

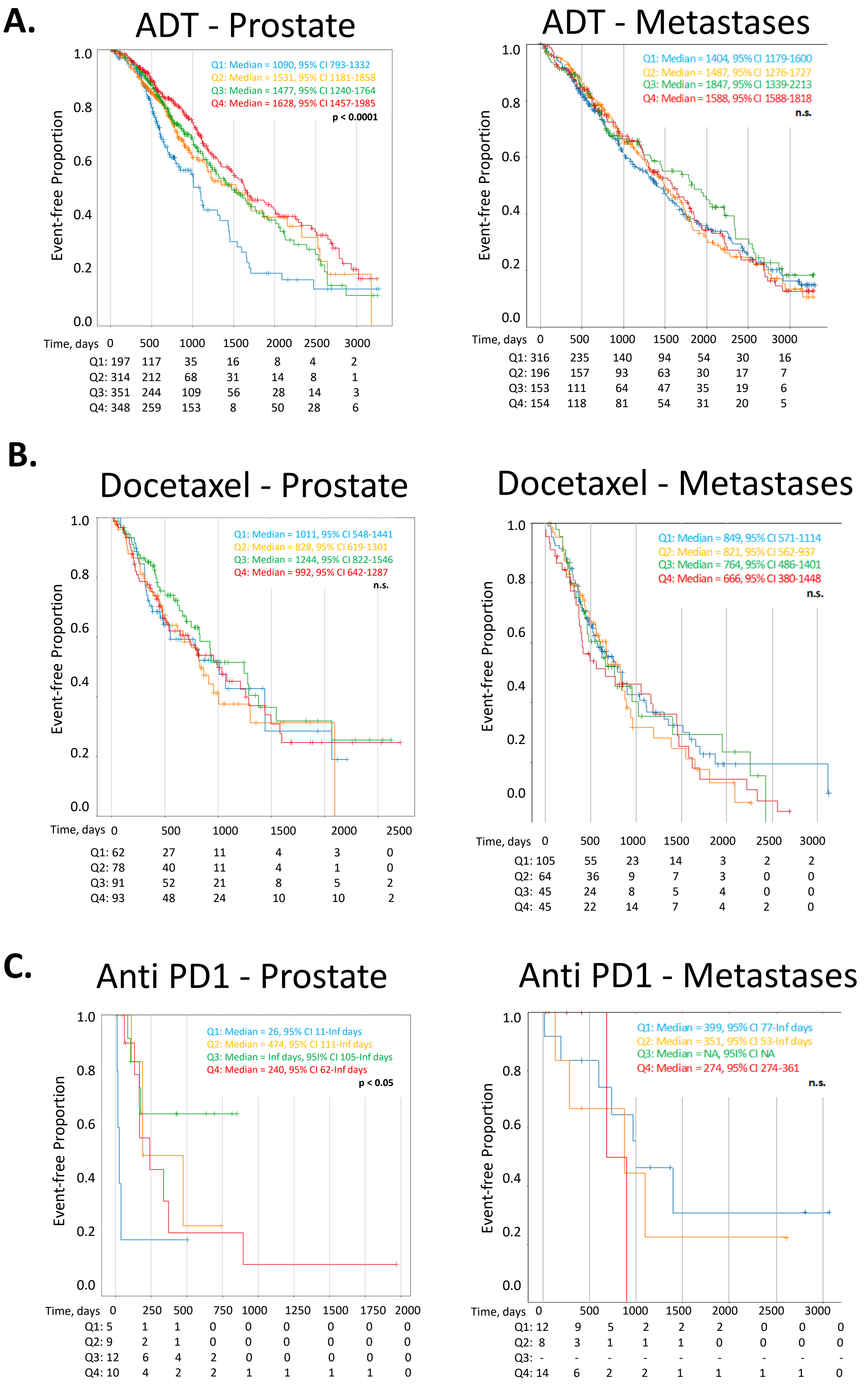
A.
ADT - Prostate
Q1: Median = 1090, 95% CI 793-1332
Q2: Median = 1531, 95% CI 1181-1858
Q3: Median = 1477, 95% CI 1240-1764
Q4: Median = 1628, 95% CI 1457-1985
p < 0.0001
Event-free Proportion
Time, days 0 500 1000 1500 2000 2500 3000
Q1: 197 117 35 16 8 4 2
Q2: 314 212 68 31 14 8 1
Q3: 351 244 109 56 28 14 3
Q4: 348 259 153 8 50 28 6
ADT - Metastases
Q1: Median = 1404, 95% CI 1179-1600
Q2: Median = 1487, 95% CI 1276-1727
Q3: Median = 1847, 95% CI 1339-2213
Q4: Median = 1588, 95% CI 1588-1818
n.s.
Event-free Proportion
Time, days 0 500 1000 1500 2000 2500 3000
Q1: 316 235 140 94 54 30 16
Q2: 196 157 93 63 30 17 7
Q3: 153 111 64 47 35 19 6
Q4: 154 118 81 54 31 20 5
B.
Docetaxel - Prostate
Q1: Median = 1011, 95% CI 548-1441
Q2: Median = 828, 95% CI 619-1301
Q3: Median = 1244, 95% CI 822-1546
Q4: Median = 992, 95% CI 642-1287
n.s.
Event-free Proportion
Time, days 0 500 1000 1500 2000 2500
Q1: 62 27 11 4 3 0
Q2: 78 40 11 4 1 0
Q3: 91 52 21 8 5 2
Q4: 93 48 24 10 10 2
Docetaxel - Metastases
Q1: Median = 849, 95% CI 571-1114
Q2: Median = 821, 95% CI 562-937
Q3: Median = 764, 95% CI 486-1401
Q4: Median = 666, 95% CI 380-1448
n.s.
Event-free Proportion
Time, days 0 500 1000 1500 2000 2500 3000
Q1: 105 55 23 14 3 2 2
Q2: 64 36 9 7 3 0 0
Q3: 45 24 8 5 4 0 0
Q4: 45 22 14 7 4 2 0
C.
Anti PD1 - Prostate
Q1: Median = 26, 95% CI 11-Inf days
Q2: Median = 474, 95% CI 111-Inf days
Q3: Median = Inf days, 95I% CI 105-Inf days
Q4: Median = 240, 95% CI 62-Inf days
p < 0.05
Event-free Proportion
Time, days 0 250 500 750 1000 1250 1500 1750 2000
Q1: 5 1 1 0 0 0 0 0 0
Q2: 9 2 1 0 0 0 0 0 0
Q3: 12 6 4 2 0 0 0 0 0
Q4: 10 4 2 2 1 1 1 1 0
Anti PD1 - Metastases
Q1: Median = 399, 95% CI 77-Inf days
Q2: Median = 351, 95% CI 53-Inf days
Q3: Median = NA, 95I% CI NA
Q4: Median = 274, 95% CI 274-361
n.s.
Event-free Proportion
Time, days 0 500 1000 1500 2000 2500 3000
Q1: 12 9 5 2 2 2 0 0 0
Q2: 8 3 1 1 1 0 0 0 0
Q3: - - - - - - - - -
Q4: 14 6 2 2 1 1 1 1 0

Figure S1
