# Supplementary figures and images for: Natural Killer Cell Infiltration in Prostate Cancers Predict Improved Patient Outcomes
Source: Prostate Cancer Prostatic Dis. 2024 Feb 28;28(1):129–37. doi: 10.1038/s41391-024-00797-0 (PMC11349934; doi:10.1038/s41391-024-00797-0)

**A.**

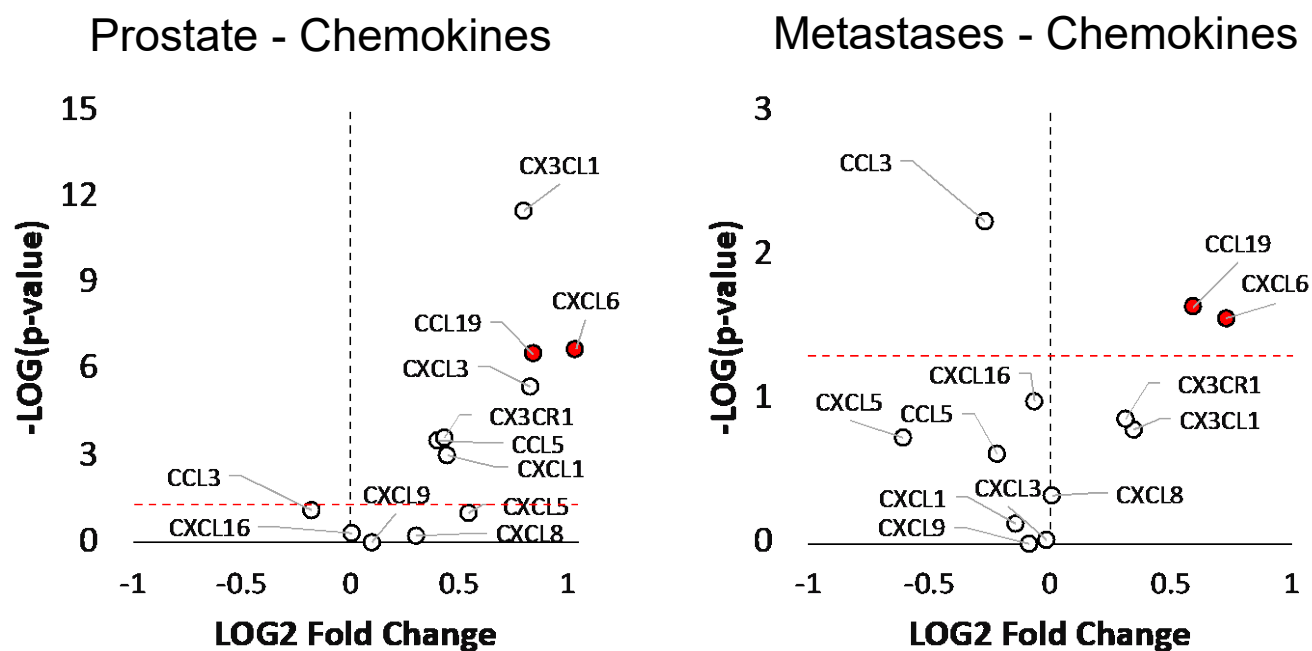

**B.**

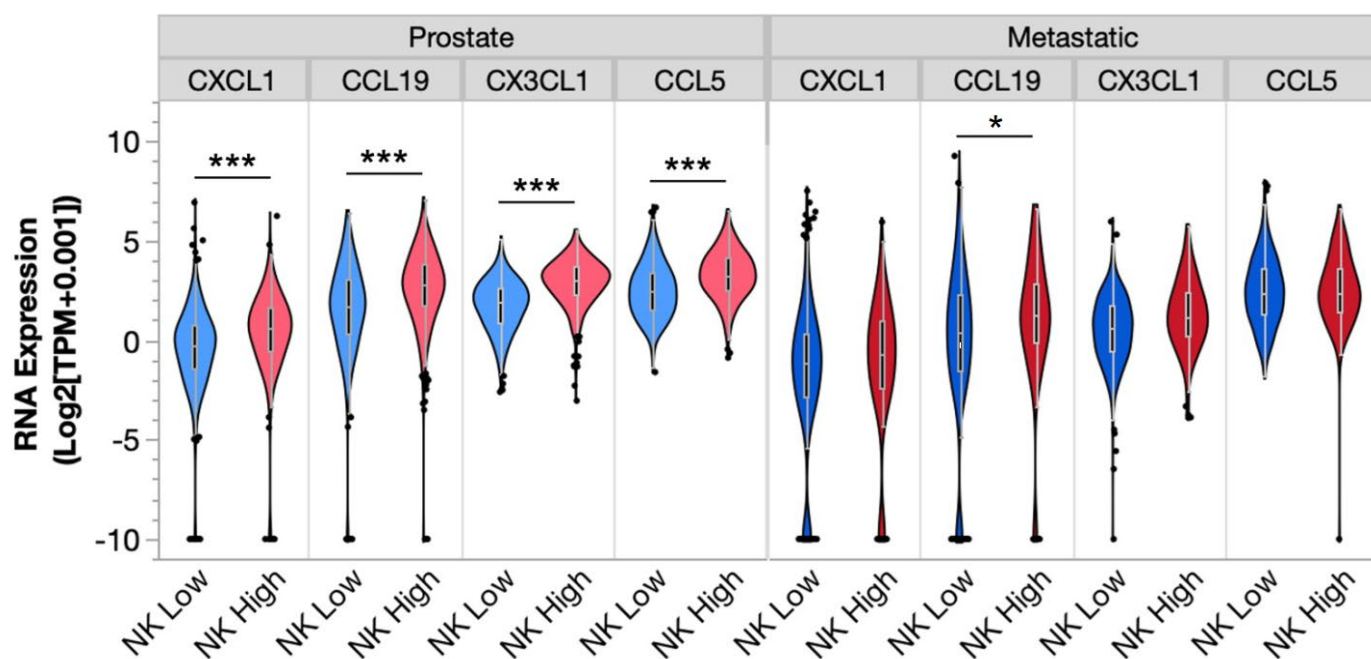

Figure S2

Supplement: Supplementary file 2 — Supplemental Figure 2 [file 41391_2024_797_MOESM2_ESM.pdf]
