# Supplementary material for: Natural Killer Cell Infiltration in Prostate Cancers Predict Improved Patient Outcomes
Source: Prostate Cancer Prostatic Dis. 2024 Feb 28;28(1):129–37. doi: 10.1038/s41391-024-00797-0 (PMC11349934; doi:10.1038/s41391-024-00797-0)
Supplement: Supplementary file 6 — Supplemental Figure Legends [file 41391_2024_797_MOESM6_ESM.docx]

**Figure S1. NK cell profiles association with concurrent PCa therapies.** Overall survival of patients with tumors from the prostate and metastasis across Q1-Q4 after treated with androgen deprivation therapies (ADT) (Abiraterone, Degarelix, Enzalutamide, Goserelin, Leuprolide, and Triptorelin) **(A)**, Docetaxel **(B)**, or Immunotherapies (Avelumab, Nivolumab, and Pembrolizumab) **(C)**. Cox proportional hazard ratios were calculated for each comparison group with significance determined as *p* values of <0.05 using log-rank statistics.

**Figure S2. NK cells and chemokine regulation. (A)** Tumor biopsy sample expression of NK cell-specific chemokines in prostate and metastatic tumors relative to NK cell high versus low tumors. The red dots indicate specific chemokines that are enriched in both samples obtained from the prostate and metastatic sites. *p*<0.05 denoted by dotted line. **(B)** Distribution of chemokines from prostate and metastatic tumors shown in violin plots in which the boundary of the violin represents the range of all data points and the white dots represent the medians.

**Table S1. Patient demographics.** Available patient data provided in CODEai and used for analysis in this study. Age is provided within a range based on 5-year increments and is further grouped by biopsy site (primary prostate or metastatic) and quartile of NK cell infiltration.

**Table S2. Gene Set Enrichment Analysis (GSEA) analysis of Hallmark pathways.** GSEA analysis was conducted based on the transcriptional profiles of tumors of the prostate or metastatic sites. The significant pathways (FDR < 0.05) are shown and the Normalized Enrichment Scores (NES) are depicted.

**Table S3. Differential gene expression profiles for primary prostate and metastatic biopsy samples.** Full dataset for differential gene expression profiles utilized for GSEA analysis are included.
